# Correction: Design principles of spectroscopic probes for biological applications

**DOI:** 10.1039/c6sc90059c

**Published:** 2016-09-02

**Authors:** Jin Zhou, Huimin Ma

**Affiliations:** a Beijing National Laboratory for Molecular Sciences, Key Laboratory of Analytical Chemistry for Living Biosystems, Institute of Chemistry, Chinese Academy of Sciences Beijing 100190 China mahm@iccas.ac.cn

## Abstract

Correction for ‘Design principles of spectroscopic probes for biological applications’ by Jin Zhou *et al.*, *Chem. Sci.*, 2016, DOI: 10.1039/c6sc02500e.

The word “invariants” in the last sentence in the Section 3.3 should be corrected as “variants” so as to read:

In fact, absorbance is measured against a corresponding reagent blank, which already eliminates the influences from some common variants that are encountered in fluorescence.^34^

The Royal Society of Chemistry apologises for these errors and any consequent inconvenience to authors and readers.

## Supplementary Material

